# The Floppy Eyelid Syndrome: Evaluating Lid Laxity and Its Correlation to Sleep Apnea Syndrome and Body Mass Index

**DOI:** 10.5402/2012/650892

**Published:** 2012-06-20

**Authors:** Panagiotis G. Beis, Catherine G. Brozou, Konstantinos I. Gourgoulianis, Chaido Pastaka, Dimitrios Z. Chatzoulis, Evangelia E. Tsironi

**Affiliations:** ^1^Department of Ophthalmology, University Hospital of Larissa, University of Thessaly Medical School, Larissa, Greece; ^2^Respiratory Medicine Department, University Hospital of Larissa, University of Thessaly Medical School, Larissa, Greece

## Abstract

*Background*. The aim of this study is to present a method of lid laxity evaluation and investigate whether there is an association between floppy eyelid syndrome (FES) and body mass index (BMI) in sleep apnea syndrome (SAS) patients compared to normal subjects. *Method*. A total of 135 participants (81 patients with SAS and 54 normal subjects) had a full ophthalmologic examination. The presence of FES was estimated in relation to SAS and BMI. *Results*. The floppy eyelid was characterized “hyperelastic,” “FES stage 1 (asymptomatic),” or “FES stage 2 (symptomatic)” depending on its laxity capacity. Hyperelastic floppy eyelid in SAS patients was statistically significant (*P* < 0.05) when compared to normals. Similarly, the presence of hyperelasticity in high-BMI SAS patients was also statistically significant (*P* < 0.05) when compared to low-BMI SAS patients. Floppy eyelid syndrome was more frequent in SAS patients than in normal subjects (*P* < 0.05), but no association was found between FES and obesity (*P* > 0.05). *Conclusion*. A classification of FES is proposed based on lid laxity. In addition to this, our data suggests a clear association of hyperelasticity and FES to SAS patients but no association between obesity and FES.

## 1. Introduction

Floppy eyelid syndrome (FES) is a condition firstly described in 1981 by Culbertson and Ostler [1–7]. Several studies have enriched the original description: affected patients (men and women), in the big majority overweight, present eyelids with increased laxity that evert easily or even spontaneously during sleep. An important number of pathologies have been associated to the syndrome either concerning the lids (ptosis, lash ptosis, blepharitis, superior lid entropion, or inferior lid ectropion) or the eye itself (papillary conjunctivitis, superficial punctate keratopathy, scarring, corneal neovascularization, keratoconus, progressive epitheliopathy, or even corneal perforation) [3, 5, 7–9].

FES has also been associated to a variety of systemic diseases as obesity, hypertension, ischemic heart disease, diabetes mellitus, skin pathologies, and most commonly sleep apnea syndrome (SAS) [5, 6, 9].

The presence of a floppy eyelid in a remarkable number of SAS patients has turned this group of patients an interesting one for investigation. SAS is a serious sleep disorder characterized by abnormal pauses of breathing or instances of abnormally low breathing during sleep. Each pause may last from a few seconds to minutes and may occur up to 30 times or more per hour. SAS prevalence is 5-6% in the general population, the main problem being that most cases remain undiagnosed. Typically, the condition is more common in older people, men predominate, and women are more often affected during pregnancy [10].

The fact that SAS is generally associated to obesity and obese patients present more frequently floppy eyelids raises the question whether FES is a characteristic of obesity or of SAS patients.

The aim of this study is to investigate whether there is an association of FES and body mass index (BMI) in SAS patients and normal subjects based on our method of lid laxity evaluation.

## 2. Patients and Methods

Participants were recruited from the Respiratory Medicine Clinic of the University Hospital of Larissa assessed for suspicion of SAS. Ophthalmologic examination was realised at the Ophthalmology Clinic of the same hospital. All participants provided written informed consent, and the study was conducted according to the Declaration of Helsinki, approved by the Ethical Committee of the University Hospital.

Subjects with previous ocular history (anterior/posterior segment disease, eye trauma, eye surgery, or photocoagulation) or diabetes mellitus were excluded. The study finally included a total of 135 subjects: 81 patients with SAS (SAS group) and 54 normal subjects (normal group). The SAS group was subdivided into two groups: SAS low-BMI group (patients with BMI < 29, *n*: 22) and SAS high-BMI group (patients with BMI > 29, *n*: 59). In a similar way, the normal group was subdivided into normal low-BMI group (*n*: 33) and normal high-BMI group (*n*: 21).

Body mass index (BMI) was defined as the individual's body weight divided by the square of his/her height.

The final diagnosis of SAS was made by the Sleep Unit of the Respiratory Medicine Clinic. All subjects were screened for age, gender, and BMI, and a complete medical and ophthalmological history was taken. They underwent a full physical examination as well as a polysomnography and stayed overnight at the hospital at the Respiratory Medicine Clinic.

All subjects were examined by the same ophthalmologist blindly that proceeded to a complete ophthalmologic evaluation including eyelid, visual acuity and visual field assessment, slit lamp biomicroscopy, tonometry, gonioscopy, fundus examination, keratotopography, and endothelioscopy.

### 2.1. Eye Lid Assessment

Both upper and lower lids were assessed in terms of lateral and medial canthal laxity as well as horizontal laxity. Regarding the upper lid, its function and horizontal distraction were assessed.

#### 2.1.1. Hyperelasticity Evaluation

The upper lid was gently everted to evaluate the laxity. Eversion resulting in exposure of the tarsus that remained despite inferior gaze position for over than three (3) seconds was characterized as hyperelasticity.

#### 2.1.2. FES Evaluation

Following the same examination technique when the lid was easily but not spontaneously everted and remained everted for up to six (6) seconds despite the down gaze position of the eye or voluntary contraction of the orbicularis oculi muscle, the patient was characterized as having asymptomatic floppy eyelid stage 1.

When the eyelid was everted easily or even spontaneously, and remained remained everted for over than six (6) seconds despite the down gaze position of the eye or voluntary contraction of the orbicularis oculi muscle, the patient was characterized as having symptomatic floppy eyelid stage 2.

Stage 1 patients did not present signs of conjunctivitis or keratitis, whereas stage 2 patients presented giant papilla conjunctivitis, keratitis, and sometimes even corneal neovascularization.

### 2.2. Statistical Analysis

Categorical variables (Tables 1 and 3) were compared using the *x* ^2^-test or the Fisher's exact test (for 2 × 2 tables). Associations were expressed as OR with 95% confidence intervals. The analysis was performed using the SPSS.

## 3. Results

Demographic data of all groups are presented in Table 1.

The presence of a floppy eyelid (regardless of the stage) predominated in the SAS group (*n*: 81, 28 patients presenting FES) than in the normal subject group (*n*: 54, 9 subjects presenting FES) (*P* < 0.05) (Table 2).

Hyperelasticity being the first characteristic to be examined was present both in SAS patients and normal subjects. Comparison of the two groups showed a statistically significant difference (*P* < 0.05) (Table 3). In addition to this, both in normals and SAS patients, hyperelasticity was more frequent when BMI was over 29 (Table 3).

**Figure 1:**
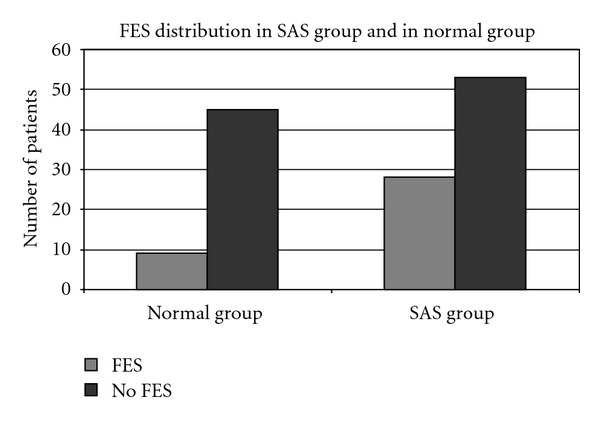
The distribution of FES both in SAS group and in normal group. We can see a predomination of subjects with FES in both groups.

When the lid laxity evaluation was taken into account in the SAS patients group (*n*: 81), there were 23 patients with stage 1 FES and 5 patients with stage 2 FES, while, in the normal subject group (*n*: 54), there were 9 subjects with stage 1 and none with stage 2. Those results were not statistically significant. Moreover, comparison of normal subjects and SAS patients regardless of the BMI value was also not statistically significant (Figure 1).

Patients that applied to either stage 1 or stage 2 of FES were calculated both for BMI < 29 and BMI > 29. The comparison between SAS patients and normal subjects did not show a statistical significant difference for either stage (Table 3).

Finally, in order to answer whether the FES BMI < 29 and BMI > 29 are a characteristic of SAS or of obesity, a comparison was made between the SAS group and the normal high-BMI group. Floppy eyelid syndrome was present in SAS patients in 35, 34% (28 of the 81 patients) and in normal obese patients in 28, 57% (6 of the 21 subjects). The difference of the two groups was not statistically significant (*P* > 0.05).

## 4. Discussion

Floppy eyelid syndrome is a clinical entity that has already been extensively investigated in terms of clinical characteristics, local and systemic associations, and treatment since its first description in 1981[1–6].

Several studies have affirmed that the loss of elastin fibers is responsible for the lax attitude of the eyelid. An important reduction is observed in the stroma of the tarsal plate in the stroma, whereas they remain mostly present around the meibomian glands. The ciliary roots being affected explain the presence of a ciliary ptosis [6]. An immunohistological study has shown an increase of elastolytic protease in the areas exhibiting less elastin compared to normals [11]. Additional mechanisms described were ischemia-reperfusion injury or repeated minor trauma or mechanical stress, provoking the same changes. In the literature, there is a discrepancy regarding lid laxity, especially the lack of a common consensus for laxity classification in FES [11]. Several authors attempted to define hyperlaxity either by the length of vertical distance between the palpebral rim and the pupil [11], horizontal distraction, snapback, vertical lid pull, horizontal distraction of the lid to the globe, proportion of the tarsal plate everted, by measuring the effort made to evert the lid and other [2].

The hallmark characteristic of FES being the eversion (spontaneous or not) was estimated to base the evaluation classification on this characteristic. The two stages of FES regarding lid laxity described above are based on the fact, that, as the disease evolves, more elastin fibers are lost. In early stages where a limited number of the fibers are lost and the lid has still some rigidity, it is evident that eversion can be realised but lasts less. On the other hand, in more advanced stages of the disease, when a larger number of elastin fibers are lost, the lid losses completely its rigidity, everts easily or even spontaneously, and may stay everted for a longer period. So a fair description of our theory may result to stage 1 for manual eversion lasting up to 6 sec and stage 2 for eversion (spontaneously or not) for over 6 sec. Further pathophysiology studies are needed to support the above-mentioned theory.

Regardless of the pathogenesis mechanism, the main characteristic of the SAS syndrome remains the increased laxity of the eyelid and its evaluation could be useful in terms of early diagnosis: many SAS cases are not diagnosed in time, and a full ophthalmologic assessment consisting of a careful lid laxity evaluation could assist to that.

The initial description [1] of the floppy eye syndrome concerned 11 obese patients, but the studies that followed reported varied associations [2, 3, 5, 6]. The results of the current study did not confirm that obese patients present the syndrome more often. Although there exists an important number of obese patients having floppy eyelids, it is evident that FES is mainly associated to SAS than obesity.

It is clear from the results of the current study that hyperelasticity is associated to obesity as it was present more frequently in overweight participants (both SAS and normals). On the other hand, the fact that FES is not associated to obesity is also confirmed in our study from the results. Comparison of obese normal subjects and obese SAS having FES did not reveal any relation between obesity and floppy eye. The same applied also when every stage of FES was examined separately.

## 5. Conclusion

The current study confirms the association of floppy eyelid syndrome and SAS as well as its association to hyperelasticity that could be considered as an initial stage of lid laxity. Our findings confirm no relationship between FES and obesity, and this is in accordance to previously published studies. An evaluation of the lid laxity is proposed based on the loss of elastin fibers that are normally responsible for the lid rigidity.

## Figures and Tables

**Table 1:** Demographic data of both groups regarding age, height, weight, and body mass index (BMI).

	SAS group (*n:* 81)	Normal group (*n:* 54)
	mean ± SD	mean ± SD
Age	49,58 ± 12,54	43,85 ± 12,52
Height	1,74 ± 0,069	1,74 ± 0,076
Weight	101 ± 19,10	86,44 ± 16,99
BMI	33,50 ± 6,26	28,56 ± 5,82

**Table 2:** The number of patients having FES is presented here for all subgroups and for all stages of FES. The number of patients without laxity is also noted to facilitate comparison. It is evident from the data that the overall number of patients presenting FES in the SAS group is greater than the one in the normal group.

	SAS group		Normal group	
	SAS low BMI	SAS high BMI	Normal low BMI	Normal high BMI
FES stage1	6	17	3	6
FES stage 2	0	5	0	0
No laxity	16	37	30	15

**Table 3:** The number of patients of each subgroup presenting hyperelasticity is presented in this table. To make the comparison easier, the number of patients without hyperelasticity is also noted. A predomination of hyperelasticity on the SAS group is clear.

	SAS		Normals	
	SAS low BMI	SAS high BMI	Normals low BMI	Normals high BMI
Hyperelasticity	8	33	5	11
No laxity	14	26	28	10
